# Functional expression and secretion of basic fibroblast growth factor in *Lactococcus lactis*

**DOI:** 10.3389/fbioe.2025.1560426

**Published:** 2025-07-24

**Authors:** Pooi Leng Ho, Yu Feng Chua, Jun Ping Quek, Say Kong Ng, Fong Tian Wong, Dave Siak-Wei Ow

**Affiliations:** ^1^Bioprocessing Technology Institute (BTI), Agency for Science, Technology and Research (A*STAR), Singapore, Singapore; ^2^Institute of Molecular and Cell Biology (IMCB), Agency for Science, Technology and Research (A*STAR), Singapore, Singapore

**Keywords:** cultivated meat, serum-free media, fibroblast growth factor 2, FGF2, Lactococcus lactis, recombinant protein expression, precision fermentation

## Abstract

**Introduction:**

Cultivated meat, produced by in vitro cell culture in bioreactors, offers a sustainable alternative to traditional meat sources. A significant challenge in its production is the high cost of mitogenic growth factors, which are essential supplements in serum-free media for cultivating meat cells. One strategy to reduce cost involves minimizing purification cost by using a food-grade host to secrete growth factors. In this study, we investigate the production of recombinant FGF2 (Fibroblast Growth Factor 2) through secretion in *Lactococcus lactis*, a Generally Recognized As Safe (GRAS) organism.

**Method:**

To enhance the secretion in *L. lactis*, we employed the USP45 secretory peptide and secretion propeptide (PP1) in the design of our recombinant FGF2-G3. Optimization was performed on various culture parameters that influence protein expression, including media formulation, nisin concentration, induction timing, temperature, and culture duration. Secreted FGF2-G3 produced under optimized conditions was purified and tested for bioactivity on Anguilla japonica pre-adipocytic cells, Aj1C-2x.

**Results and Discussion:**

We have generated a recombinant *L. lactis* strain and an optimal expression strategy to enable the production of secreted bioactive growth factors. Our results demonstrate that this system can produce FGF2 which were able to promote the proliferation of fish Anguilla japonica pre-adipocytic cells. Despite minimal purification beyond affinity purification and buffer exchange, we were able to obtain comparable specific activity to commercial FGF2. The final yields can be derived at 1.97 mg/L and through simple protein purification and buffer exchange. Finally, this study highlights the potential use of *L. lactis* secretion as an endotoxin-free alternative, compared to *E. coli*, for production of growth factors for use in cultivated meat production.

## Introduction

Cultivated meat, also known as cultured meat, has gained significant interest in recent years due to global movement towards achieving sustainability development goals. It provides a prospective alternative meat source to support demand from increasing population, reduce environmental impact from animal agriculture and avoids animal-borne diseases (Campbell et al., 2017; Godfray et al., 2018; Lynch and Pierrehumbert, 2019; Stephens et al., 2018). Cultivated meat is produced through *in vitro* culturing of animal cells in cell culture media. However, foetal bovine serum (FBS), a typical ingredient found in culture media, is derived from extracting blood serum of bovine foetuses from animal slaughter houses (Jochems et al., 2002; Kadim et al., 2015; Lee et al., 2022; Post et al., 2020; Reiss et al., 2021). This conflicts with the concept of producing meat via an animal-free approach. Thus, serum-free media formulations that are capable of sustaining cell culture were developed (Badenes et al., 2016; Das et al., 2009; Messmer et al., 2022; Skrivergaard et al., 2023; Stout et al., 2022). Subsequently, supplementation of growth factors, such as fibroblast growth factors or insulin-like growth factors, into serum-free media formulations are essential to mimic proliferative and developmental effects of FBS (Park et al., 2013; Santos et al., 2023; Venkatesan et al., 2022; Yu et al., 2023).

One such growth factor of interest is the basic fibroblast growth factor, also known as fibroblast growth factor 2 (FGF2), which is a member of the cytokine family. It acts by binding to cell surface receptors (FGFR), activating mitogenic pathways such as PI3k/Akt pathway, MAPK/ERK pathway and JNK pathway. Activation of these pathways regulates cellular responses such as growth, proliferation, migration, maintenance and differentiation (Ahmad et al., 2023; Bikfalvi et al., 1997; Yun et al., 2010). Production of recombinant growth factors, including FGF2, for supplementation into serum-free media are most frequently done in prokaryotic expression system using *Escherichia coli*. However, the recombinant proteins are produced intracellularly and has high tendency for inclusion bodies formation, which subsequently involve expensive and tedious downstream protein refolding and purification processes. Furthermore, host-cell derived impurities, in particular endotoxins, poses health risks to humans (Baneyx and Mujacic, 2004; Kaur et al., 2018; Petsch and Anspach, 2000; Sahdev et al., 2008; Thomas and Baneyx, 1996). This calls for an alternative endotoxin free expression system that is also economical for production of recombinant proteins.

*Lactococcus lactis*, a Gram-positive lactic acid bacterium that is widely used in food and therapeutic applications (Bahey-El-Din et al., 2010; Kumari et al., 2011; Song et al., 2017), presents a good alternative host for recombinant protein expression. The key feature of using *Lactococcus lactis* expression system is its ability to secrete recombinant proteins into culture medium, minimising the need for cell lysis, complex protein purification and refolding. Moreover, *L. lactis* does not produce lipopolysaccharides and has few extracellular proteases, that causes endotoxin toxicity and proteolytic degradation respectively (Frelet-Barrand, 2022; Garcia-Fruitos, 2012; Morello et al., 2008). As *L. lactis* are microaerophilic, they only require a simple static fermentation process without aeration, this makes possible for a simple and direct scale-up to industrial scale. Among the various *L. lactis* expression systems developed, the most widely used is the nisin-controlled gene expression (NICE) system, consisting of a nisRK regulatory gene integrated into bacterial host chromosome and an expression vector with nisA promoter to tightly regulate gene expression (de Ruyter et al., 1996; Mierau and Kleerebezem, 2005; Mierau et al., 2005a; Zhou et al., 2006). *L. lactis* expression system has been applied for production of several growth factor proteins (Cao et al., 2020; Gao et al., 2012; Huynh and Li, 2015; Zhou et al., 2021). In our lab, we have recently reported on valorisation of mammalian spent culture media waste to support intracellular FGF2 production in bioreactors (Rizal et al., 2024). However, comprehensive research regarding the production and secretion of functional FGF2 from *L. lactis* is not available. Hence, we set forth herein to investigate the possibility of employing *L. lactis* NICE expression system to produce and secrete biologically active FGF2. To enhance the secretion efficiency, we fused USP45 secretory peptide and secretion propeptide 1 (PP1) (Lim et al., 2017) to a thermostable FGF2 variant, FGF2-G3 (Dvorak et al., 2018). Together with optimisation of media formulation and culture conditions, we were able to obtain ∼2 mg/L of secreted FGF2-G3. Furthermore, FGF2-G3 purified from the medium was able to stimulate proliferation of the Japanese eel *Anguilla japonica* pre-adipocytic cells, comparable to commercial FGF2. Together, these results signal the potential application of *L. lactis* protein secretion system as an alternative strategy for recombinant FGF2 and potentially other growth factor production to circumvent issues faced with *E. coli* for cultured meat development.

## Materials and methods

### Bacterial strain, plasmid and cloning of FGF2-G3 gene

*L. lactis* NZ9000 and pNZ8148 plasmid (BoCa Scientific, United States) were used for cloning and expression studies. Sequence of the thermostable human FGF2-G3 was obtained from Dvorak et al. (2018). To enhance expression and secretion into medium, USP45 secretion peptide (Accession ABY84357) and propeptide 1 (PP1) (Lim et al., 2017) were fused at the *N-*terminus of FGF2-G3 sequence. For ease of purification with Ni-NTA affinity chromatography and Western blot detection, we have also included His_6_ sequence at the *N-*terminus. The nucleotide sequences corresponding to the amino acids were codon optimized and synthesized by IDT (Singapore) for expression in *L. lactis.* The coding sequence was cloned into the multiple cloning site (MCS) of pNZ8148 vector using NeBuilder HiFi Assembly (New England Biolabs, United States) (Fusion protein sequence available in Supplementary Figure S1), transformed into *L. lactis* NZ9000 and plated onto M17 agar plate containing 0.5% (w/v) glucose and 10 μg/mL chloramphenicol to screen for positive recombinant clones. The positive recombinant clones were further sequenced to ensure no mutations prior to protein expression with *L. lactis* NZ9000.

### Culture optimization for FGF2-G3 expression and secretion in *L. lactis*

Productivity of FGF2-G3 production in *L. lactis* was assessed with varying M17 media and glucose concentration. They were expressed in either M17 (supplemented with 0.5% (w/v) glucose), 2xM17 (supplemented with 0.5% (w/v) glucose) or 2xM17 (supplemented with 2% (w/v) glucose). All cultures were also supplemented with 10 μg/mL chloramphenicol for selection and maintenance of cells containing pNZ8148-FGF2-G3 plasmids. To increase production and secretion level, FGF2-G3 expression was further optimized with different nisin inducer concentrations (10, 25, 50 ng/mL), induction time points (OD_600nm_ 0.5, 1.0, 2.0), incubation temperatures (20, 25, 30, 35°C) and expression duration (4, 20 h post-induction). These parameters were investigated individually in 10 mL culture volume. The cultures were harvested by centrifugation at 3,845 *g*, 4°C for 20 min with Sorvall ST 40 centrifuge (Thermo Fisher Scientific, United States). The media fraction was aspirated and concentrated with a 10 kDa MWCO centrifugal filter (Merck Millipore, Ireland) to obtain secreted protein fraction, while the cell pellet was resuspended in PBS, incubated at 37°C for 1 h with 1 mg/mL lysozyme and 0.05 U/µL mutanolysin and then lysed via sonication. The cell lysate was centrifuged, supernatant collected as intracellular soluble fraction, and the lysed cell pellet resuspended in 6M urea to obtain intracellular insoluble fraction. All 3 fractions (secreted, soluble, insoluble) were analysed on SDS-PAGE to determine productivity level.

### Optimized production of secreted FGF2-G3

A larger production volume of FGF2-G3 was performed under optimized conditions. 250 mL of 2xM17 (supplemented with 2% (w/v) glucose and chloramphenicol 10 μg/mL) was inoculated with pre-culture and incubated at 30°C until it reaches OD_600nm_ 1.0. Nisin was then added to final concentration of 25 ng/mL to induce expression of FGF2-G3, and induction was done at 35°C for 20 h. The culture was centrifuged and the media fraction containing secreted FGF2-G3 was collected and purified via affinity chromatography.

### Purification of FGF2-G3

Media fraction containing secreted FGF2-G3 was concentrated, and buffer exchanged to buffer A (50 mM NaH2PO4, 300 mM NaCl, 10 mM Imidazole, pH 8) using crossflow filtration system. The concentrated sample was loaded onto Ni-NTA Agarose (Qiagen, United States) column and incubated for 60 min. The column was first washed with 10 mM imidazole, next with 20 mM imidazole, and finally eluted with 250 mM imidazole. The eluted fraction was buffer exchanged to PBS for protein evaluation with SDS-PAGE and bioactivity assay. The purified FGF2-G3 was quantified using Bradford method, with BSA as standard.

### Western blot and SDS-PAGE

An equal amount of protein from each fraction were run on NuPage 4%–12% Bis-Tris SDS-PAGE gel (Thermo Fisher Scientific, United States) and transferred onto nitrocellulose membrane using a Trans-Blot SD Semi-Dry Electrophoretic Transfer Cell (Bio-Rad, United States). The membrane was subsequently probed with His-Tag Antibody HRP Conjugate (Merck Millipore, United States) and detected with Clarity Western ECL Blotting Substrate (Bio-Rad, United States). Expression level of FGF2-G3 was determined by densitometric analysis of digital images using ImageJ software (National Institute of Health, United States).

To analyse purity of the large-scale purified media fraction eluted from Ni-NTA agarose column, 2 ug of protein was run on NuPage 4%–12% Bis-Tris SDS-PAGE gel (Thermo Fisher Scientific, United States) and stained with InstantBlue Coomassie Protein stain (Abcam, UK).

### Bioactivity assay of FGF2-G3

Biological activity of the purified FGF2-G3 was assessed using *Anguilla japonica* (Japanese eel) pre-adipocytic cells, Aj1C-2x (Sugii et al., 2011). The cells were cultured in Dulbecco’s modified Eagle’s medium (DMEM)/F12 media (Thermo Fisher Scientific, United States) supplemented with reduced fetal bovine serum (2.5%) and 10 ng/mL of FGF2 at 27°C with 5% CO_2_ in a humidified incubator. Cell density and viability were determined using Vi-CELL XR Cell Viability Analyzer (Beckman Coulter, United States), according to manufacturer’s instructions. Each well contains fresh DMEM/F12 medium supplemented with 2.5% FBS and varying concentrations of purified recombinant FGF2-G3 or positive control. Commercial heat stable FGF2 (PHG0368, Thermo Fisher Scientific) was used as positive control. Aj1C-2x cells were seeded into 96-well plates at seeding density of 2 x 10^4^ cells/well. After culturing for 3 days, cell viability was determined with CyQuant XTT cell viability assay (X12223, Thermo Fischer Scientific), according to manufacturer’s instructions. The absorbance reading of the media control was subtracted from the absorbance reading of each sample to determine the specific absorbance reading for each sample. Subsequently, the specific absorbance reading of each sample were normalized against the specific absorbance reading of the cells cultivated in basal media with 2.5% FBS (0 ng/mL FGF2) for Aj1C-2x cells to calculate relative absorbance fold changes. Cellular health was examined through inverted microscope under ×4 magnification (Nikon eclipse Ti with NIS-Elements AR 4.30.02 software, Nikon).

Biological activity of the purified FGF2-G3 was also further evaluated in a 6-well plate format. Aj1C-2x cells were seeded at a seeding density of 3 × 10^5^ cells/well in a 6-well plate using DMEM/F12 medium (Thermo Fisher Scientific, United States) supplemented with 2.5% FBS and varying concentrations of purified recombinant FGF2-G3 or commercial heat-stable FGF2 (PHG0368, Thermo Fisher Scientific). After 4 days of culture, cellular health was examined through inverted microscope (Nikon eclipse Ti with NIS-Elements AR 4.30.02 software, Nikon). Subsequently, cells were dissociated using TrypLE™ Express Enzyme (Thermo Fisher Scientific, United States) and viable cell density were measured using a Vi-CELL XR Cell Viability Analyzer (Beckman Coulter, United States), following the manufacturer’s protocol.

## Results and discussion

### An enhancer propeptide to enhance secretion

**FIGURE 1:**
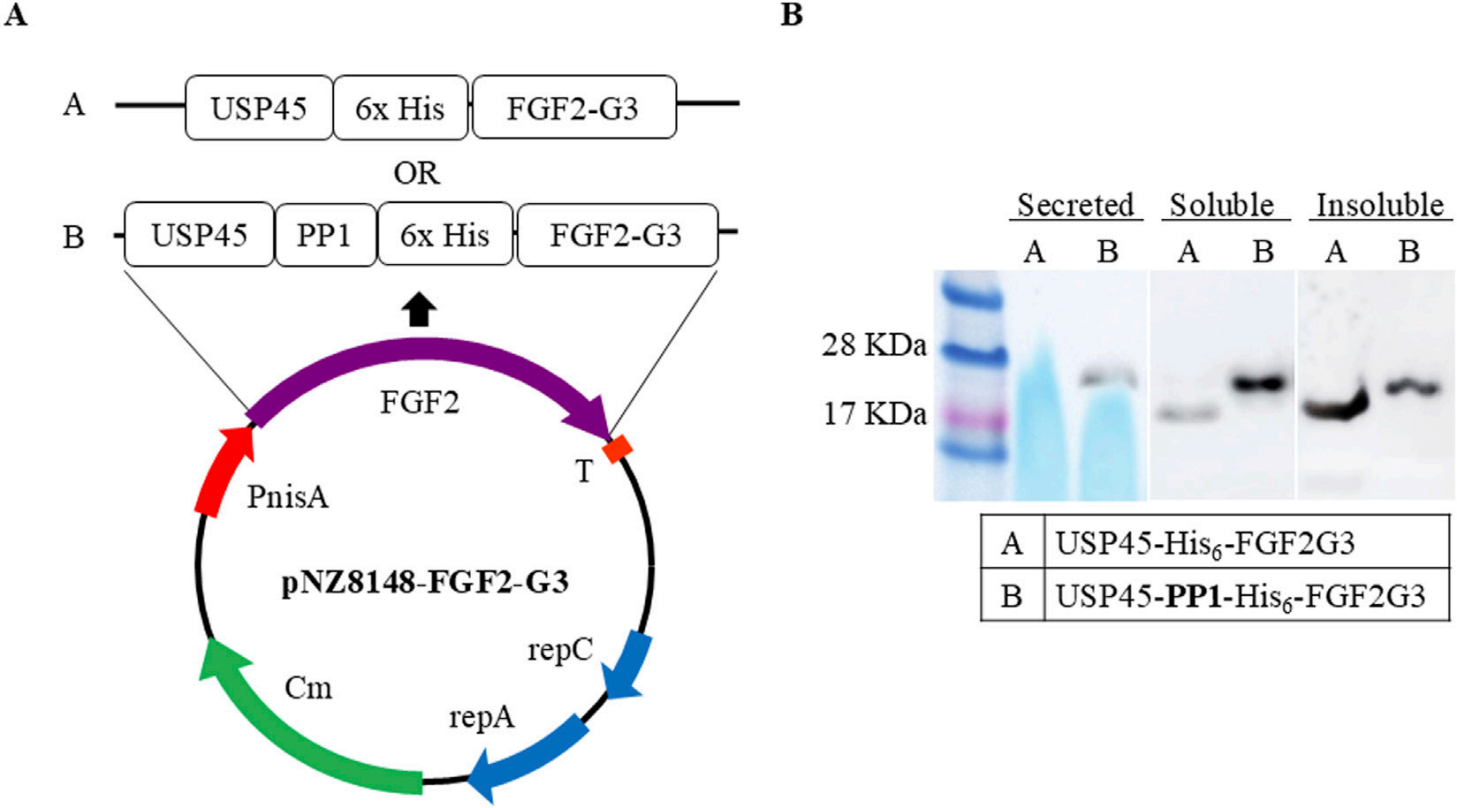
Expression vector constructs. **(A)** Schematic representation of expression vectors. FGF2-G3 with *N-*terminal fusion of signalling-secretion peptides (USP45 with and without PP1) and His_6_-tag in pNZ8148 vector. Protein sequence and insertion site available in Supplementary Figure S1 **(B)** Western blot of FGF2-G3 expression and secretion with and without propeptide (PP1) sequence in fusion plasmid.

To determine whether FGF-2 can be expressed and secreted out of *L. lactis,* we constructed an expression plasmid containing FGF2-G3, a modified and stable version that has nine amino acid mutations (Dvorak et al., 2018), with *N*-terminal fused to USP45 signal peptide. Our initial attempts to express the fusion protein resulted in low intracellular soluble yield and no protein was secreted. In previous study conducted by Lim et al. (2017), it was reported that addition of a short secretion propeptide 1 (PP1) to a USP45 fusion protein could significantly enhance protein secretion efficiency. Hence, we added PP1 between USP45 and FGF2-G3 as shown in Figure 1A.

*L. lactis* cells containing the two different recombinant plasmids (with and without PP1) were cultured in M17 media, supplemented with 0.5% glucose, and expression was induced at OD_600nm_ 0.5 with nisin at final concentration of 10 ng/mL. FGF2-G3 was allowed to be expressed for 4 h at 30°C. The expression and secretion were assessed by Western blot. Appearance of bands corresponding to two different fusion FGF2-G3 constructs (with and without PP1) were observed. It was noted that addition of PP1 not only facilitates secretion of the fusion protein, but also increases the soluble yield (Figure 1B).

### Optimisation of cultivation parameters

**FIGURE 2:**
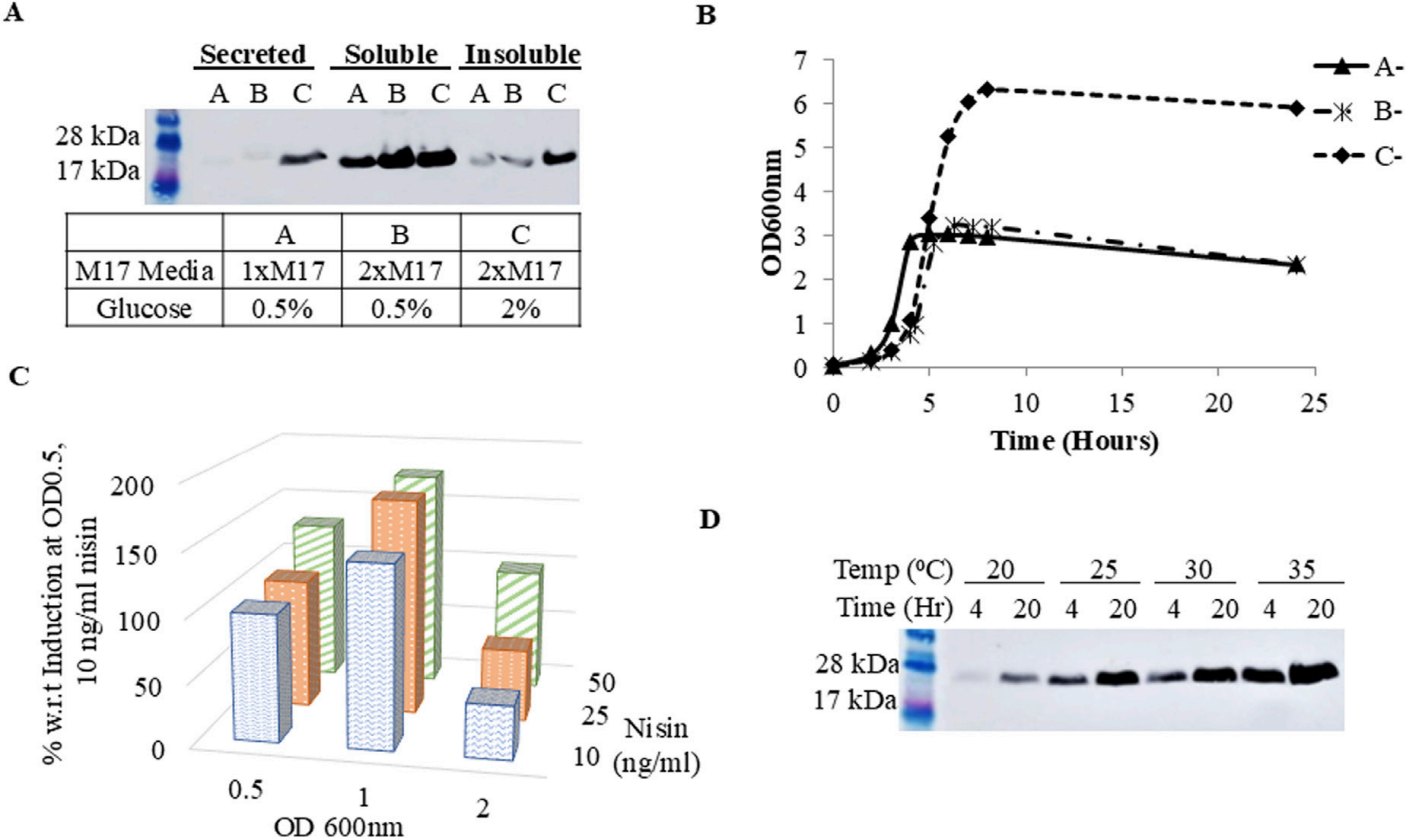
Expression of FGF2-G3 under different culture parameters. **(A)** Western blot of secreted, soluble and insoluble fraction of cell lysate. Cells were grown and induced in different media concentrations (A: M17 + 0.5% (w/v) glucose, **(B)** 2xM17 + 0.5% (w/v) glucose, **(C)** 2xM17 + 2% (w/v) glucose); **(B)** Growth curve of un-induced cells grown in different media formulations (A-: M17 + 0.5% (w/v) glucose, B-: 2xM17 + 0.5% (w/v) glucose, C-: 2xM17 + 2% (w/v) glucose) **(C)** Densitometry analysis of protein secretion yield under different induction OD (OD_600nm_ 0.5, 1.0, 2.0) and nisin concentration (10, 25 or 50 ng/mL); **(D)** Western blot of secreted protein with post-induction temperature and expression duration at 20, 25, 30 or 35°C and 4 or 20 h, respectively.

Next, optimization of culture and expression conditions were examined to increase protein secretion yield. Optimization of culture conditions began with expressing FGF2-G3 in varying M17 and glucose concentrations (M17 + 0.5% (w/v) glucose, 2xM17 + 0.5% (w/v) glucose, 2xM17 + 2% (w/v) glucose). 2xM17 + 2% (w/v) glucose medium proved to be the best for secretion of FGF2-G3 (Figure 2A).

Doubling the amount of M17 increased the soluble protein fractions. Looking at the growth curve of cells grown in different medias (Figure 2B), we hypothesize that doubling M17 not only provides additional nitrogen-based nutrients to support cell metabolism for a higher cell density culture, but also increases the buffering capacity against lactic acid produced during fermentation, due to increased amount of Disodium-β-glycerophosphate, a buffering agent found in M17 medium composition (Hayek et al., 2019; Terzaghi and Sandine, 1975; Zhang et al., 2009). These then worked in concert with increased glucose concentration, permitting *L. lactis* to extend its growth phase for higher FGF2-G3 production and, in particular, secretion titer. Further culture optimizations were performed using 2xM17 + 2% (w/v) glucose medium.

FGF2-G3 production also improved when the culture was induced at a higher cell density of OD_600nm_ 1.0 (Figure 2C). This is anticipated since higher cell density would also mean more plasmids available for induction, which in-turn raises protein expression titer. With the increase in cell density, more nisin may be needed for complete induction and thus we proceeded to determine new optimal nisin concentration. Culture induction was performed when cells reached OD_600nm_ 1.0 with nisin at final concentrations of 10, 25 or 50 ng/mL. The expression level of FGF2-G3 increased when nisin concentration was up from 10 to 25 ng/mL (Figure 2C), indicating correlation between cell density and nisin concentration needed for maximal induction. There was no further increase in expression level with 50 ng/mL nisin. Induction was also tested at OD_600nm_ 2.0, but it did not lead to higher FGF2-G3 yield.

As the FGF2-G3 used in this study has been engineered for stability, we predicted that translation and folding within *L. lactis* is not limiting, rather the rate of translation/transcription can be further improved via fermentation optimisation. Temperature and expression duration are two important post-induction conditions for optimization as they balance between bacterial growth, functional protein yield and protein degradation. It has been suggested that lower temperature improves proper protein folding and solubility (Mierau et al., 2005; Sahdev et al., 2008; Yu et al., 2021), and prolonged expression should also be avoided as it leads to higher tendency for protein degradation caused by protein instability, lactate accumulation that disrupts energy metabolism for protein expression and/or triggering of cell stress response (Zhou et al., 2006). Interestingly, results for the present study showed an increase in secreted protein yield along with increasing temperature and expression duration, with 35°C and 20 h being the optimal temperature and harvest time-point (Figure 2D).

**FIGURE 3:**
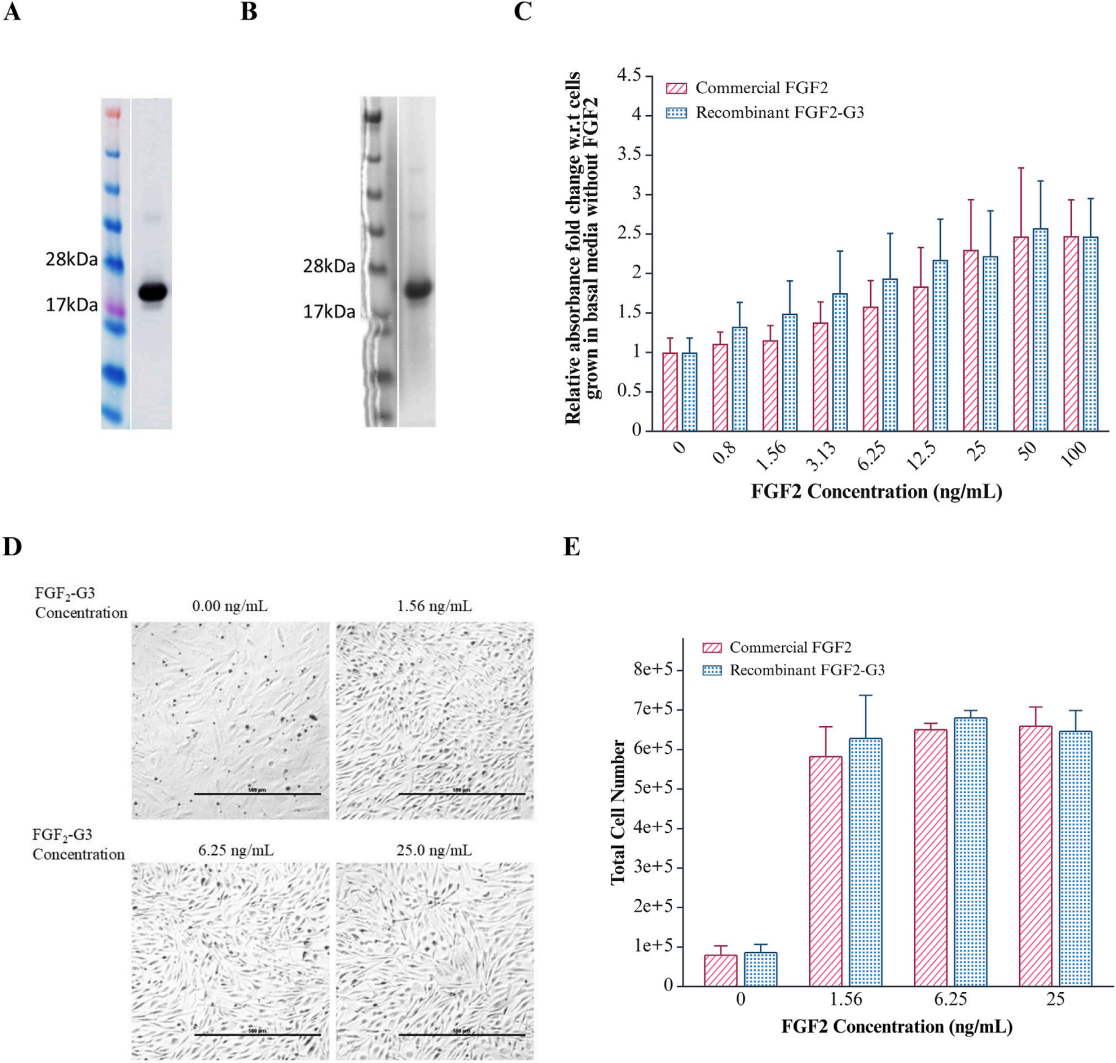
Purification and Effect of FGF2-G3 on proliferation of *Anguilla japonica* cells, Aj1C-2x. **(A)** Western blot analysis of purified FGF2-G3; **(B)** Coomassie stain of purified FGF2-G3; **(C)** XTT assay to determine cell proliferation effect of varying concentrations of commercial FGF2 and purified recombinant FGF2-G3 on Aj1C-2x cells. Absorbance readings were normalized to cells grown in medium without FGF2. Data plotted as average absorbance with error bars representing SD calculated for biological and technical triplicates. T-test analysis indicates no significant differences between commercial and recombinant FGF2 (P > 0.05); **(D)** Representative images of Anguilla japonica cells, Aj1C-2x, observed under ×4 magnification, grown in fresh DMEM/F12 medium supplemented with 2.5% FBS with varying concentrations of purified recombinant FGF2-G3; **(E)** Total cell number measured after 4 days of culture using Vi-CELL XR Cell Viability Analyzer. Data are presented as total cell counts, with error bars representing the standard deviation from biological duplicates.

The highest expression and secretion of FGF2-G3 was achieved when cells were cultured in 2xM17 medium supplemented with 2% (w/v) glucose and induced at OD_600nm_ 1.0 with 25 ng/mL nisin for 20 h at 35°C. The culture was scaled up from 10 mL to 250 mL for subsequent purification and bioactivity testing. The secreted fraction of the overexpressed FGF2-G3 was purified using immobilized metal affinity chromatography, which utilized Ni-NTA resin matrix, and the identity and purity were determined with Western blot and Coomassie staining (Figures 3A,B). The purified yield achieved herein is about 1.97 mg for 1 L of fermentation media, which is comparable to previous study by Rizal et al. (2024) where 2.6 mg/L of intracellular FGF2-G3 was produced from bioreactor fermentation. Based on an estimated requirement of 50 ng/mL recombinant FGF2-G3 in cultivated meat culture, secreted growth factors produced from 1 L fermentation provides enough growth factors for 39.4 L of cultivated meat culture. Secretion titers could be further raised with i) plasmid modifications, such as replacing promoters, ii) increasing membrane porosity by addition of chemicals, such as peptides and detergents, into culture media and iii) using bioreactor fermentation with controlled environment.

### Biological activity assessment of the purified FGF2

To assess the biological activity of the purified FGF2-G3, growth stimulation on *Anguilla japonica* (Japanese eel) pre-adipocytic cells, Aj1C-2x, was measured using XTT assay and compared against a commercial heat stable FGF2 (positive control). As shown in Figure 3C, an increase in metabolic activity was detected when cells were cultured with the purified FGF2-G3, indicating its ability to promote cell proliferation and exhibited comparable bioactivity profile to the commercial FGF2. This is further supported by an increase in cell density observed through microscopy and cell count using a cell viability analyzer (Figures 3D,E). The positive result suggests that our secreted FGF2-G3 can be used to stimulate fish stem cells for cultivated fish meat and cultivating adipocytes (fats) for enhancement of meat texture and flavour. Future bioactivity testing can be performed on mammalian cell lines, such as bovine, porcine and chicken muscle cells, to widen its application range in cultivated meat.

## Conclusion

In summary, this study demonstrated that functional FGF2 can be expressed and secreted using the *L. lactis* expression system. We employed a multi-modal optimization strategy that included a secretion-enhancing propeptide and further cultivation optimizations. Specifically, we utilized a nutrient-rich 2xM17 medium containing 2% (w/v) glucose. The ability of the *L. lactis-*produced FGF2-G3 to stimulate growth of Japanese eel cells suggests that *L. lactis* could be used as a safer alternative for production of growth factors for cultured meat development. The secretion of recombinant proteins into culture medium by *L. lactis* simplifies the production process and presents opportunities to lower production cost for cultured meat. Future efforts could be directed at expressing other growth factors, such as EGF, IGF and TGFβ1, in *L. lactis* and scaling-up in bioreactors for precision fermentation of recombinant growth factors.

## Data Availability

The original contributions presented in the study are included in the article/Supplementary Material, further inquiries can be directed to the corresponding authors.

## Abbreviations

EGF, Epidermal Growth Factor; FBS, Foetal Bovine Serum; FGF2, Fibroblast Growth Factor 2; GRAS, Generally Recognized As Safe; IGF, Insulin-like Growth Factor; NICE, Nisin Controlled Gene Expression System; TGF-β1, Transforming Growth Factor β1.
