# Supplementary figures and images for: Functional expression and secretion of basic fibroblast growth factor in Lactococcus lactis
Source: Front Bioeng Biotechnol. 2025 Jul 24;13:1560426. doi: 10.3389/fbioe.2025.1560426 (PMC12328364; doi:10.3389/fbioe.2025.1560426)

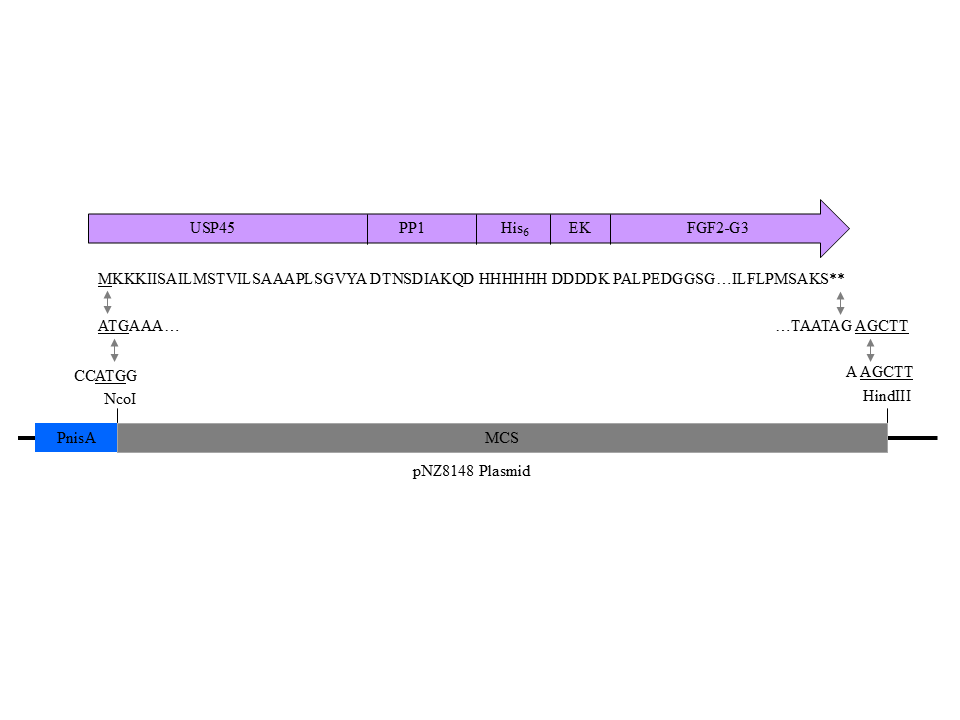

Supplement: Supplementary file 1 [file Image1.tif]
